# Role of Prefrontal Cortex on Recognition Memory Deficits in Rats following 6-OHDA-Induced *Locus Coeruleus* Lesion

**DOI:** 10.1155/2020/8324565

**Published:** 2020-07-11

**Authors:** Tuane Bazanella Sampaio, Naiani Ferreira Marques, Luisa Bandeira Binder, Carla Inês Tasca, Rui Daniel Prediger

**Affiliations:** ^1^Departamento de Farmacologia, Centro de Ciências Biológicas, Universidade Federal de Santa Catarina, Florianópolis 88049-900, Brazil; ^2^Departamento de Farmacologia, Centro de Ciências da Saúde, Universidade Federal de Santa Maria, Santa Maria 97105-900, Brazil; ^3^Departamento de Bioquímica, Centro de Ciências Biológicas, Universidade Federal de Santa Catarina, Florianópolis 88040-900, Brazil

## Abstract

Degeneration of the *locus coeruleus* (LC), the main source of cerebral noradrenaline (NA), has been reported in diverse neurodegenerative diseases, including Parkinson's diseases (PD). There is increasing evidence indicating the role of NA deficiency in the prefrontal cortex (PFC) and the development of early cognitive impairments in PD. Here, we evaluated whether a selective noradrenergic lesion of LC caused by 6-hydroxydopamine (6-OHDA) may induce memory deficits and neurochemical alterations in the PFC. Adult male Wistar rats received stereotaxic bilateral injections of 6-OHDA (5 *μ*g/2 *μ*l) into the LC, and two stainless-steel guide cannulas were implanted in the PFC. The SHAM group received just vehicle. To induce a selective noradrenergic lesion, animals received nomifensine (10 mg/kg), a dopamine transporter blocker, one hour before surgery. 6-OHDA-lesioned rats displayed impairments of the short- and long-term object recognition memory associated to reduced content of tyrosine hydroxylase in the LC. Neurochemical analysis revealed an altered mitochondrial membrane potential in LC. Regarding the PFC, an increased ROS production, cell membrane damage, and mitochondrial membrane potential disruption were observed. Remarkably, bilateral NA (1 *μ*g/0.2 *μ*l) infusion into the PFC restored the recognition memory deficits in LC-lesioned rats. These findings indicate that a selective noradrenergic LC lesion induced by 6-OHDA deregulates a noradrenergic network in the PFC, which could be involved in the early memory impairments observed in nondemented PD patients.

## 1. Introduction

Parkinson's disease (PD) is the second more prevalent neurodegenerative disease in the world. Classically, PD is characterized by the appearance of cardinal motor symptoms, such as postural instability, bradykinesia, tremor, and rigidity that are related to dopaminergic damage in the nigrostriatal pathway [1, 2]. Despite these motor impairments, PD patients also exhibited a constellation of nonmotor symptoms, which are still poorly understood and underdiagnosed [3].

Among the nonmotor symptoms, cognitive deficits are commonly observed in nondemented PD patients [4]. Initial cognitive dysfunctions in PD involve frontal-executive deficits that impact attention and executive functions and decrease them. Such changes can advance for poor performance in prefrontal-dependent tasks, visuospatial skills, and working memory, reaching to dementia linked with posterior-cortical alterations [4–7]. In addition, mild cognitive impairment is found in carriers of nondemented PD since the early stages of disease [4, 7].

PD staging was characterized by Braak et al. [8] through correlation of clinical symptoms and of Lewy body depositions. In this sense, histological identification of Lewy bodies is found in the *locus coeruleus* (LC), the main noradrenaline (NA) source in the brain [9], of PD carriers in the stage 2, previously to this pathology to reach dopaminergic structures [8, 10, 11]. Actually, the degeneration of LC observed in *postmortem* studies usually is more extensive than those found at the substantia nigra of PD patients [12–14].

There is growing evidence supporting the involvement of nondopaminergic pathways in PD progression, suggesting that the nonmotor preclinical phase can begin more than 20 years before the motor impairment appearance [3]. In this context emerges the role of LC and its importance for noradrenergic transmission in the central nervous system. LC, located in the dorsolateral pontine tegmentum, is essential for multiple brain functions including arousal, attention, higher cognitive functions, and memory [15, 16]. Although the LC had been considered a homogeneous nucleus during many years, nowadays, there is evidence supporting its anatomical and functional heterogeneity [17–19].

In particular, the prefrontal cortex (PFC) receives extensive projections from LC, which display an elevated expression of excitatory proteins and limited control by presynaptic noradrenergic *α*_2_ receptor [17, 20]. Even though several studies demonstrate that the LC lesion induced by different neurotoxins causes mnemonic impairments [21–24], the involvement of PFC in these deficits remains unclear. On the other hand, the induction of a noradrenergic lesion in the PFC of rats and monkeys revealed the essential role of NA in the PFC for adequate cognitive performance [25–28].

Thus, considering that PD patients show early degeneration of LC [12, 13] and cognitive deficits [4] and that the release of NA from LC to PFC has a critical role for cognitive functions [17], this study is aimed at investigating the involvement of PFC in the mnemonic impairment promoted by 6-OHDA-induced LC lesion in rats.

## 2. Materials and Methods

### 2.1. Animals

Adult male Wistar rats (3 months old, 280–320 g) obtained from the animal facility of the Federal University of Santa Catarina (UFSC, Brazil) were kept in an appropriate animal room on a 12 h light/12 h dark cycle (lights on at 7:00 a.m.), at a room temperature of 22 ± 2°C. Rats were housed in plastic cages (41 × 34 × 16) in groups of 3 animals with food and water ad libitum. All experimental procedures involving the animals were performed in accordance with the National Institute of Health Guide for the Care and Use of Laboratory Animals (NIH Publications, 8th edition, 2011) and were designed to minimize suffering and limit the number of animals used. The experiments were performed after the approval of the protocol by the local Institutional Ethics Committee for Animal Research (CEUA/UFSC PP830).

### 2.2. Experimental Design

To investigate whether LC lesion is able to induce cognitive dysfunction in rats, the animals were subjected to a stereotaxic surgery for bilateral injections of 6-OHDA (5 *μ*g/2 *μ*l) into the LC. One hour before, rats received an injection of nomifensine (10 mg/kg, i.p.) to protect the dopaminergic terminals [22, 29]. The SHAM group received just vehicle (saline solution containing 0.02% of ascorbic acid). The mnemonic function of animals was addressed in the object recognition task (ORT) from 12 to 15 days after the 6-OHDA injection. After the behavioral tests, animals were killed by decapitation and the LC and PFC were dissected for Western blot and neurochemical analyses.

Lastly, aiming at further addressing the role of NA in the 6-OHDA-induced memory impairments, NA replacement into the PFC was carried out. For this, the animals were submitted to stereotaxic surgery for induction of LC lesion and implantation of two stainless steel guide cannulas in the PFC. On the 14^th^ day after the surgery, bilateral saline (0.2 *μ*l/hem) or NA infusions (L-norepinephrine bitartrate salt monohydrate, 1 *μ*g/0.2 *μ*l, Sigma-Aldrich Co., St. Louis/USA) into the PFC were performed immediately before of the training sessions of ORT. Infusions were carried out at a 0.2 *μ*l/min rate using a Hamilton 10 *μ*l syringe attached by a polyethylene catheter (PE10) to a 26-gauge needle (13.1 mm long). The needle was left in place for an additional 30 s after drug infusion completion to allow the drug diffusion.

### 2.3. Stereotaxic Surgery

All animals were administered i.p. with nomifensine maleate (10 mg/kg; 1 ml/kg) (Santa Cruz Biotechnology, Santa Cruz, CA, USA)—a dopamine reuptake inhibitor—60 min before surgery, in order to protect dopaminergic terminals from 6-OHDA toxicity. After this, animals were anesthetized i.p. with ketamine (75 mg/kg; 1 ml/kg)/xylazine (8 mg/kg; 1 ml/kg) associated to local anesthesia (3% lidocaine/1 : 50.000 norepinephrine) and placed in a stereotaxic frame (Stoelting, Wood Dale, IL, USA). The scalp was shaved and swabbed with iodine, and an incision was made along the midline of the scalp exposing the bregma. Animals received 6-OHDA (5 *μ*g/2 *μ*l) (Sigma-Aldrich Co., St. Louis/USA) in a saline solution containing 0.02% of ascorbic acid or 2 *μ*l of vehicle into the LC (-9.9 mm posterior, ±1.4 mm lateral, and -7.0 mm ventral with respect to the bregma) [30]. Injections were carried out at 1 *μ*l/min, using a Hamilton 10 *μ*l syringe attached by a PE10 to a 26-gauge needle. Following injection, the needle was left in place for 3 min before being retracted, to allow complete diffusion of the medium. Additionally, two stainless steel guide cannulas (length = 11.0 mm; outer diameter = 0.6 mm) were bilaterally implanted aiming at the PFC following the coordinates (+2.7 mm anterior, ±0.5 mm lateral, and -1.7 mm ventral) [30]. Guide cannulas were fixed to the skull with acrylic resin and two stainless steel screws. To reduce the incidence of occlusion, we introduced one stylet inside each guide cannula.

### 2.4. Behavioral Tests

The behavioral experiments were carried out from 12 to 15 days after the 6-OHDA injection. All tests were performed in the light cycle, and they were scored by the same rater in an observation sound-attenuated room under low-intensity light (12 lx), where the rats had been habituated for at least 1 h before the beginning of the tests. Behavior was monitored through a video camera positioned above the apparatuses, and the videos were later analyzed with the ANY Maze® video tracking system (Stoelting Co., Wood Dale, IL, USA). A blind experimenter performed all test scoring. The apparatuses were cleaned with 10% ethanol between animals to avoid odor cues.

#### 2.4.1. Object Recognition Task (ORT)

The short- and long-term recognition memories (STM and LTM) were addressed in the ORT, performed as previously described by Ennaceur and Delacour [31] with minor modifications from Sampaio et al. [32]. Two days before testing (12 and 13 days after the 6-OHDA injection), all rats were allowed to explore the test box for 15 min once a day. The habituation phase is aimed at reducing stress, anxiety, and environmental exploration of animals on the test day. The training and testing phases occurred 24 h after the last habituation day (14 days after the 6-OHDA injection), during 3 min each, separated by an interval of 30 min or 24 h to evaluate the STM and LTM, respectively. In the training phase, the rats were exposed to two identical objects (A1 and A2) for 3 min. These objects were fixed in opposite corners 20 cm away from walls and 60 cm apart from each other. In the test phase, rats were exposed for 3 min to one of the familiar objects, and the other was replaced by a new object (B) or (C), which had a similar shape and size with different colors. The time spent by animals investigating each object in both phases was recorded. The results were expressed as % of time spent by the animals exploring the familiar and the novel objects.

### 2.5. Western Blot Analysis

LC samples were mechanically homogenized in Tris 50 mM, EDTA 1 mM, NaF 100 mM, PMSF 0.1 mM, Na3VO4 2 mM, Triton X-100 1%, glycerol 10%, and protein inhibitor cocktail (Sigma-Aldrich Co., St. Louis/USA). Lysates were centrifuged (3,000 rpm for 10 min; at 4°C) to eliminate cellular debris. Protein content was determined by the method of Lowry et al. [33], using bovine serum albumin (BSA) as the standard. The supernatants were diluted to a final protein concentration of 3 *μ*g/ml in SDS-PAGE buffer. The samples (60 *μ*g of protein) and prestained molecular weight standard (GE Healthcare Life Sciences, MA, USA) were separated on 12% resolving with 4% SDS-polyacrylamide electrophoresis gels. After electrotransfer to PVDF membrane, it was blocked with 3% BSA solution. The blot was incubated overnight at 4°C with anti-tyrosine hydroxylase (Abcam, Cambridge, MA, USA). In the sequence, the membrane was washed and incubated with horseradish peroxidase-conjugated secondary antibody (Upstate Cell Signaling, SP, Brazil) for 1 h at room temperature and developed with enhanced luminol-based chemiluminescent (Merck KGaA, Darmstadt, Germany). Anti-*β*-actin (Upstate Cell Signaling, SP, Brazil) was stained as a protein loading control. Relative optical density (O.D.) of the western blotting bands was quantified using ImageJ (NIH, Bethesda, MD, USA) software for Windows. Each value was derived from the ratio between arbitrary units obtained by the protein band and the respective *β*-actin band. The results were shown as % of control.

### 2.6. Neurochemical Analyses

After the behavioral evaluation, animals were euthanized by decapitation and the whole brain was quickly removed and LC and PFC were rapidly dissected in ice-cold KREBS ringer buffer (KRB) (122 mM NaCl, 3 mM KCl, 1.2 mM MgSO_4_, 1.3 mM CaCl_2_, 0.4 mM KH_2_PO_4_, 25 mM NaHCO_3_, and 10 mM D-glucose, bubbled with 95% CO_2_/5% O_2_ up to pH 7.4). Slices (0.4 mm) were prepared using a McIlwain Tissue Chopper (The Mickle Laboratory Engineering Co. Ltd., England) and separated in KRB at 4°C. After sectioning, slices were incubated in KRB for 30 minutes, at 37°C, for metabolic recovering [32].

#### 2.6.1. Reactive Oxygen Species (ROS) Generation

Slices from LC and PFC were incubated with molecular probe 2,7-dichlorodihydrofluorescein diacetate (DCFH-DA, Sigma-Aldrich Co., St Louis, MO, USA), which diffuses through the cell membrane and is hydrolyzed by intracellular esterases to the nonfluorescent form dichlorodihydrofluorescein (DCFH). DCFH reacts with intracellular ROS to form dichlorofluorescein (DCF), a green fluorescent dye. DCF fluorescence intensity is proportional to the amount of ROS. Brain slices were incubated with 500 *μ*l of DCFH-DA (80 *μ*M) for 30 min at 37°C and then washed and kept in KRB. Fluorescence was measured in a fluorescence microplate reader (TECAN) in one slice from brain regions, assayed in triplicates, using excitation and emission wavelengths of 480 and 525 nm, respectively [34].

#### 2.6.2. Propidium Iodide (PI) Incorporation

Cell damage was addressed by evaluating the uptake of the fluorescent exclusion dye, PI, which is a polar compound that enters only cells with damage membranes. LC and PFC slices were prepared and incubated with 7 *μ*g/ml of PI for 30 min at 37°C. After washes with KRB, the fluorescence was quantified by a microplate reader (TECAN). Inside the cells, PI complexes with DNA and emits an intense red fluorescence (630 nm) when excited by green light (495 nm) [35].

#### 2.6.3. Mitochondrial Membrane Potential

Mitochondrial membrane potential was measured by incubation with the molecular probe tetramethylrhodamine ethyl ester (TMRE, Sigma-Aldrich Co., St Louis, MO, USA) (100 nM) for 30 min at 37°C [36]. Fluorescence was measured using wavelengths of excitation and emission of 550 and 590 nm, respectively. Results were obtained as relative fluorescence units (RFU) from individual experiments. RFU variation among the experiments was normalized by expressing data as the means and SEM of percentage relative to controls.

#### 2.6.4. Protein Measurement

Protein content was measured, using bovine serum albumin (Sigma-Aldrich Co, MO, USA) as standard [33].

### 2.7. Statistical Analysis

The normal distribution of the data was tested with D'Agostino and Pearson normality tests. Data were analyzed by Student's *t-*test. The results are presented as the mean + standard error of mean (SEM). Probability values less than 0.05 (*p* < 0.05) were considered as statistically significant. Statistical analysis was performed using the GraphPad Prism 8.0 (GraphPad, San Diego, CA, USA).

## 3. Results

### 3.1. Effects of LC Lesion Induced by 6-OHDA on the Cognitive Function of Rats

**Figure 1:**
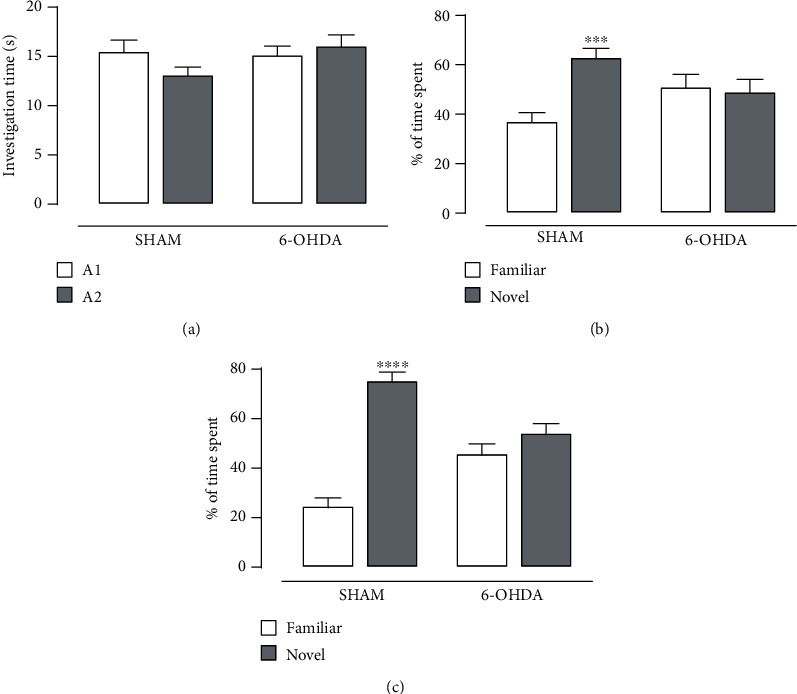
6-OHDA-induced LC lesion promotes recognition memory impairment in rats. Effect of 6-OHDA (5 *μ*g/hem) into the LC on the percentage of time spent by the animals exploring the familiar and the novel objects in the ORT. (a) Investigation time in the training session. (b) Short- and (c) long-term object recognition memories. Data are shown as the means + SEM of 10 animals per group. ^∗∗∗^*p* < 0.001 and ^∗∗∗∗^*p* < 0.0001 compared to the familiar object in the same group (Student's *t*-test).

From 12 to 15 days after LC lesion induced by 6-OHDA, cognitive functions of animals were evaluated in the ORT. In the training session, the two identical objects (A1 and A2) used were equally explored, demonstrating that there was not object preference by animals (Figure 1(a)). Moreover, Student's *t*-test revealed lack of significant differences between the percentage of time spent by the animals exploring the familiar and the novel objects from the 6-OHDA group during the test session evaluated 30 min (*t*_(18)_ = 0.273, *p* = 0.788) or 24 h (*t*_(18)_ = 1.299, *p* = 0.210) after the training session, indicating a mnemonic impairment caused by 6-OHDA-induced LC lesion (Figures 1(b) and 1(c)).

### 3.2. Effects of 6-OHDA Injection into the LC on the Tyrosine Hydroxylase Levels in the LC

**Figure 2:**
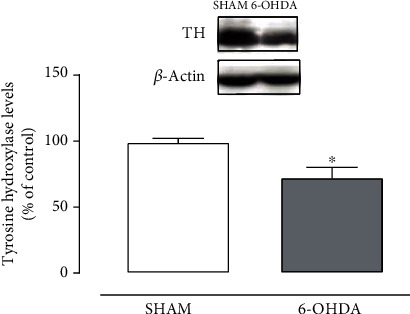
6-OHDA injection into the LC promotes reduced levels of tyrosine hydroxylase in the LC of rats. Effect of 6-OHDA injection into the LC on the tyrosine hydroxylase levels in the LC at 15 days after administration. Data are expressed as the means + SEM of 4 animals per group. ^∗^*p* < 0.05 (Student's *t*-test).

To confirm the LC lesion, tyrosine hydroxylase levels in the LC were carried out by Western blot. Student's *t-*test revealed that 6-OHDA-lesioned rats display decreased levels of tyrosine hydroxylase in the LC when compared with the SHAM group (*t*_(6)_ = 2.981, *p* = 0.025) (Figure 2).

### 3.3. Effects of LC Lesion Induced by 6-OHDA on Neurochemical Parameters in LC and PFC Slices

**Figure 3:**
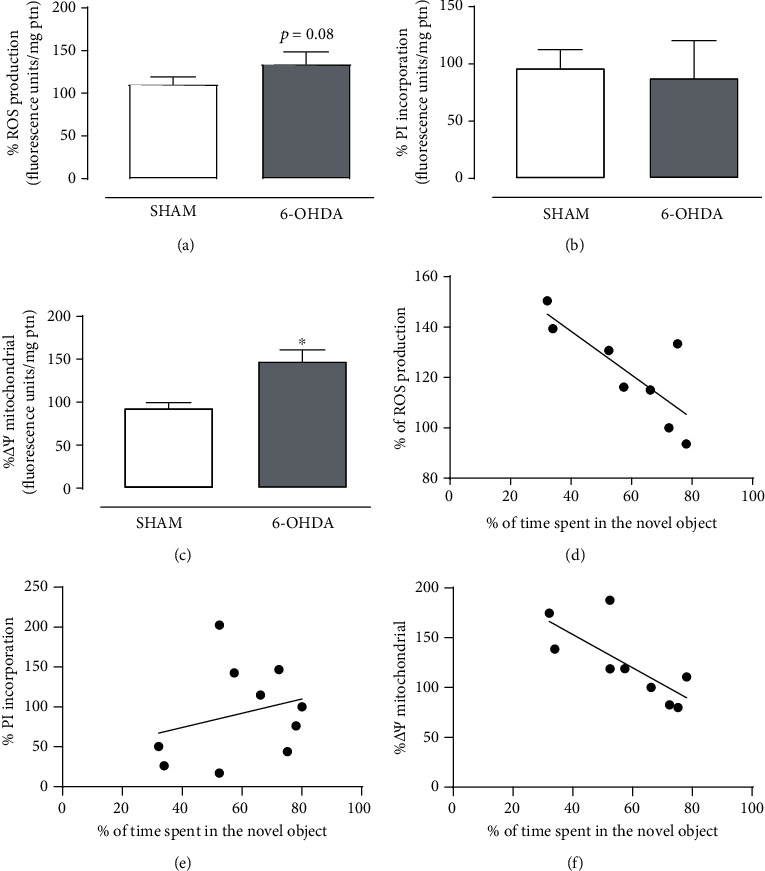
6-OHDA-induced LC lesion promotes mitochondrial membrane disruption in the LC of rats. Effect of 6-OHDA-induced LC lesion on (a) ROS production, (b) cell membrane damage, and (c) mitochondrial membrane potential in the LC slices at 15 days after administration. Data are expressed as the means + SEM of 4-5 animals per group. ^∗^*p* < 0.05 (Student's *t*-test). Correlation between the % of time spent in the novel object during the long-term memory test and the (d) ROS production, (e) PI incorporation, and (f) mitochondrial membrane potential in the LC. Each dot represents one pair comprising both analyses from the same animal.

To investigate the involvement of PFC in the mnemonic impairment promoted by 6-OHDA-induced LC lesion, slices from LC and PFC were subjected to neurochemical evaluation. LC slices from 6-OHDA-lesioned rats showed altered mitochondrial membrane potential (*t*_(7)_ = 3.125, *p* = 0.017) (Figure 3(c)) and a negative correlation between the % of time spent in the novel objection during the long-term memory session and the mitochondrial membrane potential (*r* = −0.7495, *p* = 0.020), indicating that an increased mitochondrial membrane potential is related to a poor performance in the ORT (Figure 3(f)). On the other hand, no changes were observed in the cell membrane integrity (*t*_(8)_ = 0.212, *p* = 0.837) and oxidative stress (*t*_(6)_ = 2.071, *p* = 0.083) levels (Figures 3(a) and 3(b)). However, a negative correlation between the % of time spent in the novel objection during the long-term memory session and the ROS production was found in the LC (*r* = −0.7924, *p* = 0.019) (Figure 3(d)). No correlation was observed between the behavioral data and the PI incorporation in this area (*r* = 0.2582, *p* = 0.4714) (Figure 3(e)).

**Figure 4:**
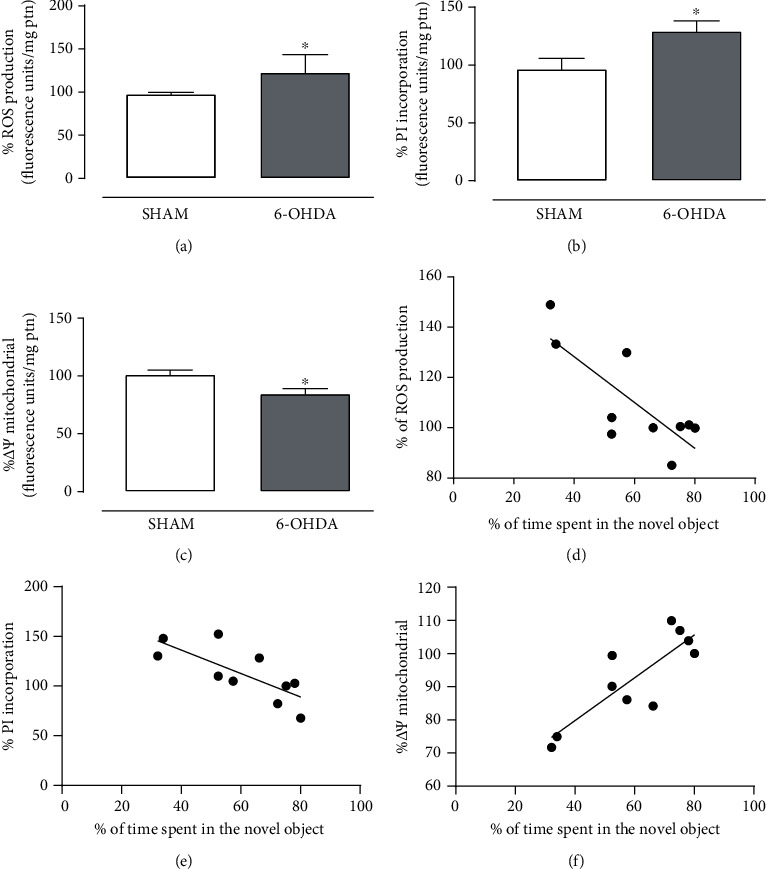
6-OHDA-induced LC lesion promotes oxidative stress, mitochondrial membrane disruption, and cell membrane damage in the PFC of rats. Effect of 6-OHDA-induced LC lesion on (a) ROS production, (b) cell membrane damage, and (c) mitochondrial membrane potential in the PFC slices at 15 days after administration. Data are expressed as the means + SEM of 4-5 animals per group. ^∗^*p* < 0.05 (Student's *t*-test). Correlation between the % of time spent in the novel object during the long-term memory test and the (d) ROS production, (e) PI incorporation, and (f) mitochondrial membrane potential in the PFC. Each dot represents one pair comprising both analysis from the same animal.

Importantly, PFC slices from the 6-OHDA group exhibited cell membrane damage (*t*_(8)_ = 2.346, *p* = 0.047), increased ROS production (*t*_(8)_ = 2.527, *p* = 0.035) and mitochondrial membrane potential disruption (*t*_(8)_ = 2.439, *p* = 0.041) when compared to the SHAM group (Figures 4(a)–4(c)), suggesting that the lesion of LC neurons is associated with neurochemical changes in the PFC. Additionally, the correlation analysis corroborates these data, demonstrating a negative correlation between the % of time spent in the novel objection during the long-term memory session and the oxidative stress production (*r* = −0.7880, *p* = 0.007) and PI incorporation (*r* = −0.7569, *p* = 0.011) (Figures 4(d) and 4(e)). Also, a positive correlation was found between the behavioral data and the mitochondrial membrane potential (*r* = 0.8413, *p* = 0.002) (Figure 4(f)).

### 3.4. Effects of NA Administration into PFC on the Recognition Memory Deficits Promoted by 6-OHDA-Induced LC Lesion

**Figure 5:**
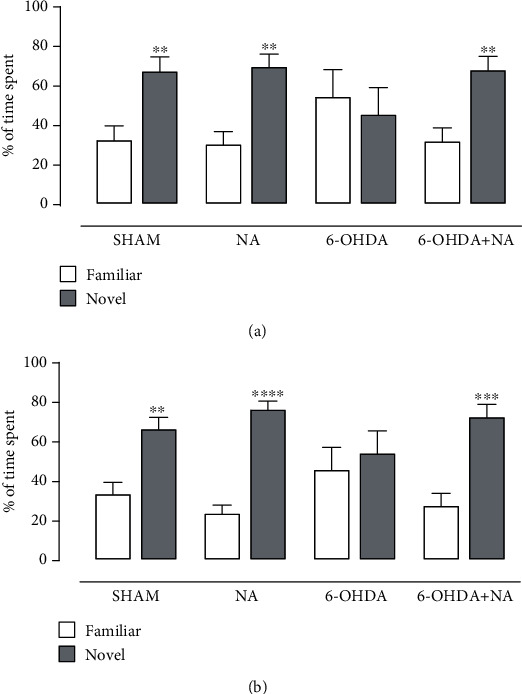
NA replacement into the PFC restores the 6-OHDA-induced mnemonic impairments. Effect of NA replacement (1 *μ*g/hem) in the PFC on the (a) short- and (b) long-term memory impairments induced by LC lesion addressed in the ORT. Data are expressed as the means + SEM of 8-10 animals per group. ^∗∗^*p* < 0.01, ^∗∗∗^*p* < 0.001, and ^∗∗∗∗^*p* < 0.0001 compared to the familiar object in the same group (Student's *t*-test).

To evaluate the putative involvement of noradrenergic dysfunction in the PFC on the mnemonic impairment caused by 6-OHDA-induced LC lesion, a NA replacement was carried out in this brain area. As observed in Figure 5, NA administration in the PFC restored both short- and long-term memories (short-term: *t*_(16)_ = 3.663, *p* = 0.0021; long-term: *t*_(16)_ = 4.885, *p* = 0.0002) disrupted by 6-OHDA-induced LC lesion (short-term: *t*_(16)_ = 0.463, *p* = 0.650; long-term: *t*_(16)_ = 0.522, *p* = 0.609).

## 4. Discussion

The present findings suggest the involvement of noradrenergic neurotransmission in the PFC on recognition memory impairments induced by LC lesion in rats. 6-OHDA-induced LC lesion caused short- and long-term recognition memory impairments addressed in the ORT associated to decreased levels of tyrosine hydroxylase in the LC. Neurochemical analysis revealed an altered mitochondrial membrane potential and an oxidative stress correlation in LC-lesioned rats. Regarding the PFC, an increased ROS production, cell membrane damage, and mitochondrial membrane potential disruption were observed. Also, NA replacement into the PFC reversed the impaired mnemonic functions elicited by LC lesion in rats.

LC is the main source of NA in the central nervous system of mammals [9]. Through its projections, LC modulates the cortical, subcortical, and brainstem circuits impacting in several functions, such sensory, motor, and cognitive [22, 37]. Interestingly, LC damage has been reported in diverse neurodegenerative diseases, which may represent a triggering and/or progression event of neurodegenerative process [38]. For instance, the degeneration of LC precedes the appearance of classical pathological hallmarks in the dopaminergic nigrostriatal pathway in PD [12, 14].

Corroborating the notion of the LC role on the cognitive process, we demonstrated that the 6-OHDA injection into the LC decreases the tyrosine hydroxylase levels, confirming the 6-OHDA-induced LC lesion, and promotes short- and long-term recognition memory disruptions assessed at two weeks after the surgery. Recently, our research group reported that this LC lesion model causes cognitive impairments after 7 and 21 days of 6-OHDA injection into the LC [22]. Taken together, these observations might be related to the involvement of LC degeneration on the mild cognitive impairment found in nondemented patients during the early stages of neurodegenerative diseases.

6-OHDA is considered a neurotoxin classically used as the model of PD [39]. Its mechanism of action is based on the oxidative stress induction. 6-OHDA accumulates in the cytosol of catecholaminergic neurons where it undergoes a nonenzymatic autooxidation. Additionally, 6-OHDA is able to inhibit the activity of the electron transport chain [40]. These two neurotoxic mechanisms culminate in the oxidative stress exacerbation [39], which is a relevant neurochemical event in PD pathogenesis [41].

It has been demonstrated that 6-OHDA can activate different biochemical and neuronal death pathways [42]. We found that 6-OHDA-lesioned rats display reduced levels of tyrosine hydroxylase and altered mitochondrial membrane potential and a trend to increase ROS generation in slices from LC. In addition, a negative correlation between oxidative stress in the LC and mnemonic impairment 24 h after the training session was found, indicating that an increased ROS content is related to poor performance in the ORT. However, PI incorporation was not altered by 6-OHDA-induced LC lesion. Once that PI is useful to stain necrotic cells [43], this result seems to corroborate our recent findings showing the main involvement of apoptosis mechanisms in the 6-OHDA-induced LC damage associated to NA reduction of about 50% in this area [22].

Thus, 6-OHDA administration into the LC caused mitochondrial disruption in the LC and mnemonic decline addressed in the ORT. Since PFC modulates the attention, learning, and the skill to explore and recognize a novelty [44], we investigated the consequences of LC lesion on the PFC function. Herein, we demonstrated that the 6-OHDA-induced LC damage caused increased ROS production, mitochondrial membrane potential disruption, and cell damage in PFC slices. Importantly, all neurochemical analyses performed in the PFC slices were correlated to long-term recognition memory deficit observed, strengthening the importance of relationship PFC-LC for adequate cognitive function. Corroborating these findings, we recently demonstrated that LC lesion induced by 6-OHDA approximately doubled the levels of NA metabolite and NA turnover in the PFC, suggesting an increased NA metabolism and its consequent depletion in the synaptic cleft [22].

In order to address the role of noradrenergic PFC dysfunction in the recognition memory deficits of LC-lesioned rats, we microinjected NA directly into the PFC. NA replacement was able to restore both the short- and long-term memory impairments. Dayan and Yu [45] demonstrated that increased LC activity facilitates the attention, focus, and performance from an exploratory situation to targeted task. On the other hand, dorsal and ventral frontoparietal areas are important to discern an event with potential behavioral significance [46]. Moreover, PFC receives neuronal fibers from LC more excitable than those designed to other cortical areas and NA and dopamine are coreleased from LC and are crucial for cognitive function [17, 47].

It must be conceded that additional studies are necessary to elucidate the exact target associated to observed effects after the NA administration into PFC. In fact, NA has high affinity for all types of adrenergic receptors (*α*_1_, *α*_2_, *β*_1_, *β*_2_, and *β*_3_ receptors), being that all are expressed in the PFC [48]. For instance, studies with laboratory animals and humans demonstrate that *α*_2A_ receptors agonist are able to improve PFC function [49–51]. On the other hand, Grzelka et al. [52] reported that NA evokes depolarization dependent on *β*_1_- and not *α*_1_ or *α*_2_ receptor stimulation. In addition, our previous study suggests the involvement of *β*_3_ receptor in the NA restorative effect [22].

In conclusion, the present findings showing that LC lesion induces recognition memory deficits linked, at least in part, to noradrenergic dysfunction in the PFC allow us to speculate that similar alterations could be involved in the mild cognitive impairments observed at early stages of PD.

## Data Availability

Data will be made available on request.
